# Repetitive Transcranial Magnetic Stimulation, Cognition, and Multiple Sclerosis: An Overview

**DOI:** 10.1155/2018/8584653

**Published:** 2018-01-18

**Authors:** Grigorios Nasios, Lambros Messinis, Efthimios Dardiotis, Panagiotis Papathanasopoulos

**Affiliations:** ^1^Department of Speech and Language Therapy, Higher Educational Institute of Epirus, Ioannina, Greece; ^2^Department of Neurology, Neuropsychology Section, University of Patras Medical School, 26504 Patras, Greece; ^3^Department of Neurology, University of Thessaly Medical School, Larisa, Greece; ^4^Department of Neurology, University of Patras Medical School, 26504 Patras, Greece

## Abstract

Multiple sclerosis (MS) affects cognition in the majority of patients. A major aspect of the disease is brain volume loss (BVL), present in all phases and types (relapsing and progressive) of the disease and linked to both motor and cognitive disabilities. Due to the lack of effective pharmacological treatments for cognition, cognitive rehabilitation and other nonpharmacological interventions such as repetitive transcranial magnetic stimulation (rTMS) have recently emerged and their potential role in functional connectivity is studied. With recently developed advanced neuroimaging and neurophysiological techniques, changes related to alterations of the brain's functional connectivity can be detected. In this overview, we focus on the brain's functional reorganization in MS, theoretical and practical aspects of rTMS utilization in humans, and its potential therapeutic role in treating cognitively impaired MS patients.

## 1. What Is Multiple Sclerosis (MS)?

Multiple sclerosis (MS) is an autoimmune, chronic central nervous system disease of unknown etiology, presenting as an ongoing demyelinating, inflammatory, and degenerative process, affecting both grey and white matters of the brain and the spinal cord, and resulting in the accumulation over the years of disabling motor and cognitive handicaps. Quality of life; personal, social, and professional status; and life expectancy are all significantly challenged by the disease [1–3].

One of its most puzzling characteristics is the subclinical phase prior to diagnosis, which could last for years and subtly affect cognitive aspects of nervous system functioning. Indeed, there is evidence suggesting that deterioration of cognitive performance could be detected years (even decades) before formal diagnosis [4, 5]. Unfortunately, there are currently no validated biomarkers to preliminary track the neuroimmunological phenomena underlying this subclinically active disease phase [6].

Additionally, patients which are initially diagnosed with a radiologically isolated or clinically isolated syndrome (RIS or CIS) which years later progress to definite MS forms have only recently been targeted with disease-modifying medications during the initial phase, resulting in an overall large number of patients worldwide in whom treatment initiation comes rather late in the disease course. This disappointing fact, resulting perhaps in the accumulation of disability in the majority of MS patients over the years (especially after the 1st or 2nd decade of the disease course), may be linked to the continuing and increasing CNS lesion load and tissue damage and has fortunately forced specialists in the field to become alert of the notion that “time is brain” and that “effective intervention during a limited period early in the course of MS is critical for maintaining neurological function and preventing subsequent disability” [7].

## 2. Cognition in MS

The cognitive aspect of MS was not recognized widely until the last two decades, although Charcot has described it as part of its clinical picture almost 150 years ago [8]. Now we know that 40–70% of all MS patients do have cognitive impairment which affects their lives [2, 9]. Even in the so-called “benign” form of the disease, 15 years after the diagnosis with an EDDS score remaining considerably low (up to 3), cognitive disorders can be diagnosed in half of these patients [10]. The database PubMed which was accessed on 15 May 2017, with keywords “cognition in Multiple sclerosis” revealed 2256 items, 1698 of them (75.26%) were published after 2006.

Another major aspect of the disease is brain volume loss (BVL), which is present in all phases and all types (relapsing and progressive) of the disease, and it is linked to both motor and cognitive disabilities [11–13]. BVL is widespread in both white and grey matter tissues and cortical and subcortical structures. Among other sites, thalamic damage is directly related to cognitive deficits in all forms of the disease, even in clinically isolated syndromes (CIS) [14, 15]. Moreover, while white matter atrophy is 3-fold compared to that in healthy controls and remains 3-fold during the course of the disease, gray matter atrophy is initially 3-fold in CIS patients compared to healthy controls but increases to 14-fold in SPMS patients [16, 17].

The deterioration of cognitive performance, usually subtle at least during the first years of the disease course, is almost impossible to be diagnosed by routine neurological testing; therefore, special neuropsychological assessments are needed [18]. This deterioration may not be clinically evident at first and may be hidden by neuroplasticity, that is, the brain's capacity to reorganize its networks in order to keep on functioning despite tissue damage. Using state-of-the-art functional and static neuroimaging magnetic resonance imaging techniques such as fMRI and diffusion tensor imaging (DTI), we can study the brain's effort to overcome the ongoing structural damage and maintain sufficient functions and many new important insights are becoming apparent, as we will discuss them in the sections that follow.

We do not know a lot about disease-modifying medications' ability to act directly on patients' cognitive performance, or what we know is that they do not have a significant influence [19] what we suspect (and hope) is that they do so indirectly by protecting the accumulation of brain tissue damage and delaying brain volume loss. It seems that even in the small proportion of patients achieving the desired NEDA status (no evidence of disease activity) over time, cognitive deterioration was not precluded [20]. The role of cognitive rehabilitation in various central nervous system diseases and MS has recently emerged [21]. Additionally, other nonpharmacological interventions are also being discussed as having a potentially beneficial role in ameliorating physical and cognitive aspects of the disease [22]. Among these interventions, repetitive transcranial magnetic stimulation (rTMS) seems to have both the scientific and theoretical support and also evidence from experimental models of the disease and trials in patients that can play an important role in MS's management.

## 3. TMS and rTMS

Transcranial magnetic stimulation (TMS) is a neurostimulatory and neuromodulatory technique, based on the principle of electromagnetic induction of an electric field in the brain [23]. This method has behavioral consequences and therapeutic potentials. Barker et al. in 1985 described a method of directly stimulating the human motor cortex using a pulsed magnetic field [24]. During the last 2-3 decades, TMS has become a method of choice for noninvasive stimulation of the brain in conscious human subjects to study the excitability of different cortical areas and to map the connectivity of neuronal pathways [25, 26]. When TMS pulses are applied repetitively, they can modulate cortical excitability, either decreasing or increasing it, depending on the parameters of stimulation. TMS has immediate as well as after-effects on the human cortex. rTMS has local and remote effects on neural function which can be excitatory or inhibitory [27]. The direction, magnitude, and duration of conditioning rTMS effects depend on the stimulation site, frequency, intensity, and the duration of the rTMS training. For example, after-effects last longer when the number of rTMS stimuli applied is increased [28]. Low-frequency (1 Hz) rTMS given over the primary motor cortex reduces corticospinal excitability [29], but higher-frequency rTMS increases corticospinal excitability (Pascual-Leone et al., 1994 and [30]). It has also been shown that repeated rTMS is capable of evoking long-lasting cumulative plastic changes of cortical function not only in the stimulated cortex but also in the remote functionally interconnected areas that outlast the stimulation period [31]. The way rTMS acts on molecular and neuronal level is not yet well understood. It has become clear that rTMS can change structural, functional, and molecular properties of neurons, which may depend on the simultaneous conduction of action potentials. rTMS-mediated changes interfere with the ability of neurons to express distinct forms of plasticity beyond the stimulation period [30, 32]. Evidence is growing about the rTMS-induced modification of cerebral blood flow, glucose metabolism, and neuronal excitability in the stimulated area as well as in interconnected brain regions [33]. After-effects of rTMS may represent changes in synaptic efficacy known as long-term potentiation (LTP) and long-term depression (LTD). The balance between “LTP/LTD-like” phenomena, which underlie many processes happening in the brain, that is, learning and memory, is altered by rTMS. Esser et al. exploited a new approach based on combined rTMS/high-density electroencephalography (hd-EEG) providing a direct noninvasive evidence for LTP bilaterally over the premotor cortex in humans induced by rTMS [34].

TMS could possibly have additional effects such as endocrine after-effects, histotoxicity, and effects on neurotransmitters, immune system, and autonomic function, which are not yet fully understood [23]. Potential therapeutic effects of rTMS have already been explored, and “the use of TMS has grown dramatically in the past decade, new protocols of TMS have been developed, changes in the devices have been implemented, TMS is being increasingly combined with other brain imaging and neurophysiologic techniques including fMRI and EEG, and a growing number of subjects and patients are being studied with expanding numbers of longer stimulation sessions” [23].

An increasing number of trials worldwide investigated the therapeutic role of rTMS in depression, schizophrenia, addictions, posttraumatic stress disorders, pain, migraine, stroke, autism, multiple sclerosis, and neurodegenerative disorders such as Alzheimer's and Parkinson's disease [35]. Accordingly, animal studies have been employed to assess the effects of rTMS on synaptic plasticity. Among them, there are studies indicating an additional therapeutic role of electromagnetic stimulation in demyelinating processes: experimental animal models of MS (experimental autoimmune encephalomyelitis) have proven that rTMS modifies astrocytosis, cell density, and lipopolysaccharide levels, suggesting that TMS could be a promising treatment for neuroinflammatory conditions such as multiple sclerosis [36].

Sherafat et al. have shown that after inducing demyelination, using local injection of lysophosphatidylcholine within the corpus callosum of adult female Sprague-Dawley rats and then applying electromagnetic fields (EMFs) postlesionally significantly reduced the extent of the demyelinated area and increased the level of myelin basic protein staining within the lesion area, suggesting that EMFs potentiate proliferation and migration of neural stem cells and enhance the repair of myelin in the context of demyelinating conditions [37].

What is very interesting—and there is accumulating evidence towards this—is that we can affect cognitive processing in healthy humans by rTMS. Guse et al. conducted a systematic overview of high-frequency rTMS (HF-rTMS) studies assessing neurocognition in order to better understand the potential of rTMS to induce long-term effects on cognition. High-frequency rTMS (10–20 Hz) is most likely to cause significant cognitive improvement when applied over the left (dorsolateral) prefrontal cortex, within a range of 10–15 successive sessions and an individual motor threshold between 80 and 110% [38].

The correct positioning of the coil is also very important for the effects of rTMS. Localization of the stimulation site by individually fMRI-guided TMS neuronavigation, instead of using the 10–20 EEG system, results in stronger and more robust TMS effects, inducing long-lasting cognitive improvement [39]. Sato et al. designed a study by using event-related potentials (ERPs) to clarify the effect of magnetic stimulation on cognitive processing. They found that a 1.00 Hz rTMS pulse train over the left dorsolateral prefrontal cortex increased P300 latencies by 8.50 ms at Fz, 12.85 ms at Cz, and 11.25 ms at Pz. In contrast, neither 0.75 nor 0.50 Hz rTMS pulse trains over the left dorsolateral prefrontal cortex nor 1.00, 0.75, and 0.50 Hz rTMS pulse trains over the right dorsolateral prefrontal cortex altered P300 latencies. These results indicate that rTMS frequency affects cognitive processing. The authors suggested that the effects of rTMS vary according to the activity of excitatory and inhibitory neurons in the cerebral cortex [40].

Esslinger et al., using a multimodal fMRI-rTMS approach, demonstrated changes in cortical plasticity in humans during executive cognition [41]. They examined 12 healthy control subjects in a crossover study with fMRI while performing an *n*-back working memory (WM) task and a flanker task engaging cognitive control, after real and sham 5 Hz rTMS to the right dorsolateral prefrontal cortex (DLPFC). Reaction times during the *n*-back task were significantly shorter after rTMS than after sham stimulation, supporting an excitatory effect of high-frequency rTMS. Interestingly, rTMS compared with sham stimulation caused no activation changes at the stimulation site (right DLPFC) itself but significantly increased connectivity within the WM network during *n*-back and reduced activation in the anterior cingulate cortex during the flanker task. These findings show the plastic changes in prefrontal connected networks downstream of the stimulation site as the substrate of the behavioral effect [31]. Li et al. investigated the effects of high-frequency (10 Hz) rTMS applied over the left DLPFC on cognitive control of young healthy participants and explored the time course changes of cognitive processing after rTMS using event-related potentials (ERPs). A Stroop task was performed, and an electroencephalogram (EEG) was recorded. The results revealed that multiple sessions of rTMS can decrease reaction time (RT) under both congruent and incongruent conditions and also increased the amplitudes of both N2 and N450 compared with sham rTMS. This observation supports the view that high-frequency rTMS over the left DLPFC not only recruits more neural resources from the prefrontal cortex by inducing an electrophysiologic excitatory effect but also enhances efficiency of resources to deploy for conflict resolution during multiple stages of cognitive control processing in healthy young people [42]. Hsu and colleagues conducted a systematic review and meta-analysis of the literature (1990–2014) to evaluate the effects of noninvasive brain stimulation (rTMS and tDCS) on cognitive function in healthy older adults and patients with Alzheimer's disease (AD). They concluded that noninvasive brain stimulation has a positive effect on cognitive function in physiological and pathological aging [43].

## 4. Brain's Functional Reorganization in MS

As sophisticated techniques have been introduced in the near past, we are facing a new era in which neuroplasticity can be studied not only as a unique brain ability to reorganize its functional networks in order to overcome aging and diseases but also as a new therapeutic target. In fact, neuropsychological rehabilitation (neurorehabilitation), accompanied by new noninvasive neurostimulation–neuromodulation methods, is becoming popular, partially due to the lack of effective pharmacological treatments. As Maggio and Vlachos state, “understanding the role of neural plasticity under pathological conditions, novel therapeutic approaches could be designed to promote, block, or shift the balance between distinct forms of plasticity in specific brain regions and at diverse stages of pathological brain conditions” [44].

Neuroplasticity is increasingly studied as altered brain functional connectivity both at rest (resting-state functional connectivity (rs-FC)) and during tasks. Hyperconnectivity or hypoconnectivity can be detected, depending on the severity and extension of structural brain damage, the nature of disease process, and its time course. These alterations could be adaptive or maladaptive.

Particularly in multiple sclerosis, studies have shown that patients in early stages activate additional brain areas adjacent to those primarily involved during task performance, allowing patients to perform normally prior to cognitive deficits being detectable on neuropsychological assessment [45]. This additional activation serves as a compensatory mechanism allowing the individual to maintain intact cognitive functioning for a period of time, functionally compensating for injury associated with progression of the disease and thus masking defects [46, 47]. Mainero and colleagues scanned matched healthy subjects and patients with relapsing-remitting MS (RRMS) with no or only mild cognitive deficits while performing neuropsychological testing (the Paced Auditory Serial Addition Test (PASAT) and a recall task), and the relation between fMRI changes during both tasks and T2 lesion load was investigated. Patients with RRMS exhibit altered patterns of activation during tasks exploring sustained attention, information processing, and memory. During these tasks, fMRI activity was greater in patients with better cognitive function than in those with lower cognitive function. Authors concluded that functional changes in specific brain areas increase with increasing tissue damage suggesting that they may also represent adaptive mechanisms that reflect underlying neural disorganization or disinhibition, possibly associated with MS [48].

Staffen and colleagues performed a functional MRI study during PVSAT (Paced Visual Serial Addition Task), a visual analogue to PASAT (Paced Auditory Serial Addition Task), in 21 recently diagnosed RRMS patients and matched healthy controls. A group analysis of the functional imaging data during the PVSAT revealed different activation patterns for patients compared with control subjects. In healthy volunteers, the main activation was detected at the right hemispheric frontal cortex (Brodmann area 32). In patients, the main activation was detected at the right hemispheric frontal cortex (Brodmann areas 6, 8, and 9). In addition, the left hemispheric Brodmann area 39 was activated. The different patterns of activation, accompanied with intact performance in a sustained attention task of this multiple sclerosis sample compared with healthy controls, were interpreted as the consequence of compensatory mechanisms, in other words as an expression of neuronal plasticity during early stages of a chronic disease [49].

In contrast to task-based fMRI, resting-state functional connectivity (rs-FC) examines the communication between different brain regions within neural networks at “rest.” Resting-state functional connectivity (rs-FC) studies have noted that increased activation could be interpreted as either adaptive or maladaptive, depending on the progression of the disease. Increased connectivity during rs-FC is thought to serve as a compensatory mechanism for cognitive deficits early in the MS disease process [21, 50, 51], but later in the disease, these extra connections are associated with worse performance [21, 52]. Cader et al. concluded that both forms of adaptive functional change, that is, the enhancement of the coherence of interactions between brain regions normally recruited (functional enhancement) and the recruitment of alternative areas or the use of complementary cognitive strategies, could limit clinical expression of the disease and particularly of cognitive impairments [51].

MS patients, trying to compensate the ongoing structural damage, do not only activate additional cerebral areas but also change strategies, and indeed, this is partially effective. An excellent proof of this is provided in the article of Bonnet et al.: while performing a go/no-go task of increasing complexity, patients could follow the performance of healthy control subjects to a point. For the most complex condition, patients presented both collapse of additional cerebral recruitment and significant lower cognitive performance compared to controls. Authors questioned the cerebral mechanisms allowing the maintenance of normal performances in patients with RRMS according to the level of cognitive demand. They found that, “contrary to healthy subjects, patients with MS did not exhibit a correlation between cerebellar activation and better performances.” Patients' retained performance was correlated with higher activation in medial prefrontal regions (IG and CG), areas known to be involved in decision-making; in other words, they exhibit a transfer of function to cerebral areas skilled to manage controlled processes. This new medial frontal recruitment could support a functional strategy of compensation in patients with MS. In a multicenter study, significant correlations were found between abnormal fMRI patterns of activations and deactivations and behavioral measures, cognitive performance, and brain T2 and T1 lesion volumes. These results support the theory that a preserved fMRI activity of the frontal lobe is associated with a better cognitive profile in MS patients [53].

In an elegant recent study, Rocca and colleagues [54] investigated rs-FC abnormalities within the principal brain networks in a large cohort of MS patients, with various forms and stages of the disease. Connectivity abnormalities and correlations with clinical/neuropsychological/imaging measures were evaluated. MS patients showed reduced network average rs-FC versus controls in the default-mode network. At regional level, a complex pattern of decreased and increased rs-FC was found. Reduced rs-FC correlated with T2 lesions. Reduced thalamic rs-FC correlated with better neuropsychological performance, whereas for all the remaining networks, reduced FC correlated with more severe clinical/cognitive impairment. Similar findings have been reported for Alzheimer's disease, in which subjects in an early preclinical phase show relatively increased prefrontal cortical activation with memory deficits [55].

Sumowski and colleagues explored the cognitive reserve hypothesis by testing how could lifetime intellectual enrichment (estimated with vocabulary knowledge) lessen the negative impact of brain disease on cognition; in other words, patients with greater enrichment are able to withstand more severe neuropathology before suffering cognitive impairment or dementia. Multiple sclerosis patients' cerebral activity (functional magnetic resonance imaging blood oxygen level-dependent signal) and behavioral performance were recorded during the visual *n*-back working memory task. Results revealed strong positive correlations between intellectual enrichment and cerebral activity within the brain's default network, indicating that patients with greater enrichment were able to maintain resting-state activity during cognitive processing better. Furthermore, intellectual enrichment was negatively associated with prefrontal recruitment, suggesting that patients with lesser enrichment required more cerebral resources to perform the same cognitive task as patients with greater enrichment [56].

However, it is important to appreciate the complexities of interpreting differences in patterns of activation across the brains of subjects with pathology relative to healthy controls. First, fMRI identifies brain regions in which activity is associated with task performance, not those that are necessary [57]. Secondly, alternative strategies for performance of a task can be associated with differences in patterns of activation without being able to be interpreted in a simple way as adaptive [58]. Schoonheim et al. reviewed the recent functional connectivity literature in MS and the potential effects on cognition that functional connectivity changes may have [59]. A “compensatory” change is seen in the brains of MS patients in the form of both increased activation and increased connectivity. Studies investigating the “default mode network” (DMN) found increased DMN connectivity in clinically isolated syndrome (CIS) patients [60] and decreased DMN connectivity in progressive MS, which was related to cognitive impairment [61]. Which reported connectivity changes can be said to be “compensatory”? Which are “maladaptive”? Authors conclude on the requirement of “a more holistic approach, encompassing both activation and connectivity data into a frame of network dynamics in a longitudinal fashion.”

## 5. rTMS in MS

Palm et al. reviewed the application of noninvasive brain stimulation techniques for the improvement of several neurologic and psychiatric disorders in MS patients. Specifically, the efficacy of tDCS and TMS for the treatment of depressive symptoms, fatigue, tactile sensory deficit, pain, motor performance, and spasticity was assessed in several studies and showed mixed results [22].

Due to the lack of effective pharmacological treatments alone, rTMS in combination with medication has been used with significant efficacy mainly for the improvement of spasticity [62–65], fatigue and depression [22], lower urinary tract dysfunction [66], gait [67], and hand dexterity [61, 68]. Most studies however have certain methodological limitations, such as small number of participants and low-to-moderate level of efficacy, indicating the emerging need for more studies in the future. Symptoms, such as fatigue, are better targeted with tDCS [69].

## 6. rTMS for Cognition in MS

Considering the previously presented literature, we have several reasons why one should consider using rTMS to treat cognitively impaired MS patients: firstly, we do not have effective pharmacological treatments for the nearly two-thirds of all MS patients who become cognitively impaired through the disease course, and their lives are negatively influenced; secondly, there is an accumulating body of evidence that patients' brains undergo functional reorganization even from the initial disease phases, by altering functional connectivity in various regions, and this acts as a compensatory mechanism; thirdly, a growing number of MS patients are exposed to rTMS training protocols for other symptoms, without any major safety or adverse event considerations; and fourthly and more importantly, noninvasive neurostimulation techniques such as rTMS have shown beneficial effects on cognitive performance in healthy persons and in patients with various neurological diseases, by evoking neuroplasticity changes, in other words enhancing the brain's functional capacity.

Additionally, higher cognitive reserve [56] and cognitive rehabilitation interventions [70, 71] have proved effective in ameliorating cognitive performance in MS patients, and the underlying mechanism seems to be the induced neuroplasticity changes [21, 56, 72]. One could, therefore, consider using, and even combining, these available nonpharmacological, noninvasive interventions.

Despite the theoretical support of such clinical use, there is, to our knowledge, only one, recently published, study for the therapeutic use of rTMS on cognition in MS patients [73]. In this study, Hulst et al. investigated the effects of high-frequency rTMS of the right dorsolateral prefrontal cortex (DLPFC) on working memory performance, while measuring task-related brain activation and task-related brain connectivity in patients with MS. The authors reported that *n*-back task accuracy improved after applying real rTMS (and not after sham rTMS) only in patients. At baseline, MS patients, compared to healthy controls, showed higher task-related frontal activation, which disappeared after real rTMS. Task-related functional connectivity between the right DLPFC and the right caudate nucleus and bilateral (para) cingulate gyrus increased in patients after real rTMS when compared to sham stimulation. The authors interpret these results as an rTMS-induced change in network efficiency in MS patients, implicating a potential role for rTMS in cognitive rehabilitation in MS. With the limitation of the small sample of participants (17 MS patients and 11 HCs), the results of this study are very promising and of course call for more trials in order to provide more robust evidence of rTMS therapeutic effects on cognitively impaired MS patients.

## 7. Conclusions

The road that lies ahead is long, but the first steps have been made: the neurological community now recognizes that cognitive impairment is an important component of MS (with the recently introduced concept of cognitive impairment associated with multiple sclerosis (CIAMS)) [74], stipulating that cognition must be included in diagnostic, follow-up, and therapeutic evaluations. Methods to neuropsychologically assess patients with MS and suitable imaging techniques to monitor cognitive function are now more widely accessible. Functional connectivity changes in the healthy and diseased brain can be detected and modified by interventions. We must go one step further and target cognitive functions therapeutically through well-designed clinical trials, with carefully selected large numbers of suitable patients, combining neuropsychological methods and noninvasive neurostimulation–neuromodulation and neuroimaging techniques, in order to offer widely effective treatments to our patients living with MS.
